# Identification of Candidate miRNAs Associated with Shear Force and Intramuscular Fat in Pig Breeds

**DOI:** 10.3390/foods15152693

**Published:** 2026-07-30

**Authors:** Qi Zhang, Jing-Bo Zhang, Yun-Peng Zhang, Jing Xu, Suthar Teerath Kumar, Yuan Zhao, Shu-Min Zhang

**Affiliations:** 1 College of Animal Science and Technology, Key Laboratory of Animal Production, Product Quality and Security, Ministry of Education, Jilin Provincial Key Laboratory of Animal Nutrition and Feed Science, Jilin Agricultural University, Changchun 130118, China; zhang12100214@163.com (Q.Z.);; 2 Institute of Animal and Veterinary Sciences, Jilin Academy of Agricultural Sciences (Northeast Agricultural Research Center of China), Changchun 136100, China; 3 College of Animal Science and Technology, Key Laboratory of Animal Production, Product Quality and Security, Ministry of Education, Jilin Agricultural University, Changchun 130118, China

**Keywords:** meat quality, microRNA, pig breeds, intramuscular fat, tenderness

## Abstract

In this comparative study of 12 pigs (six Songlei black [SL] and six Duroc × Landrace × Yorkshire [DLY]), small RNA sequencing was performed on longissimus dorsi muscle samples to identify miRNAs associated with meat quality traits. A total of 343 known and 191 novel miRNAs were identified, including 21 differentially expressed miRNAs (DE-miRNAs) between breeds. Enrichment analysis showed that their target genes were enriched in the PI3K-Akt, MAPK, and calcium signaling pathways. Integration of miRNA and transcriptome datasets identified a regulatory network comprising five key miRNAs (ssc-miR-10383, ssc-miR-127, ssc-miR-370, ssc-miR-874, and a novel miRNA, novel_494) and 32 target genes annotated to autophagy, AMPK, mTOR, and FoxO signaling pathways. All five miRNAs were negatively correlated with IMF content (r = −0.77 to −0.95) and positively correlated with shear force (r = 0.62 to 0.76). This study provides the first miRNA expression profile of the longissimus dorsi muscle in SL and DLY pigs. These findings suggest that coordinated miRNA regulation may contribute to breed-specific differences in fat deposition and meat tenderness, providing candidate biomarkers for improving pork quality.

## 1. Introduction

Pork is one of the most widely consumed animal-derived protein sources worldwide, and its quality is a major determinant of consumer acceptance and market value [1,2]. Chinese indigenous pig breeds possess unique meat flavor, tender texture, and rich nutrition. Therefore, they serve as important germplasm resources for improving pork quality in breeding programs. By contrast, the Duroc × Landrace × Yorkshire (DLY) pig is the dominant breed in large-scale production. It exhibits fast growth and high lean meat percentage. However, its meat flavor, intramuscular fat deposition, and other traits often fall short of those seen in indigenous black pigs [3,4,5]. Therefore, understanding the molecular regulatory mechanisms that underlie meat quality differences between these two types of pigs provides important theoretical support and practical guidance for high-quality pork breeding.

Meat quality is a complex trait shaped by genetics, nutrition, and management. Understanding its molecular basis is essential for breeding programs aimed at improving pork quality. In addition to genetic factors, nutritional strategies can modulate meat quality traits, including intramuscular fat deposition and muscle fiber composition [6]. Meanwhile, recent studies have highlighted the gut–muscle axis as a key regulator of energy metabolism and meat quality in pigs [7].

Intramuscular fat (IMF) content is closely associated with meat quality attributes. In pork, higher IMF levels are generally linked to improved juiciness, enhanced flavor, and greater consumer acceptability [8]. The composition and spatial distribution of IMF also influence meat processing properties, water-holding capacity, and commercial value [9]. Shear force is a widely used objective measure of tenderness and is strongly correlated with the sensory perception of meat softness and chewiness [10]. The deposition of intramuscular fat (IMF) is governed by a dynamic balance between adipogenic differentiation, lipid synthesis and hydrolysis, and the metabolic environment of skeletal muscle [11]. Shear force, in turn, reflects the combined effects of perimysial and endomysial collagen architecture, sarcomere shortening, post-mortem proteolysis via the calpain/calpastatin system, glycolytic pH decline, and fiber-type composition, all of which are modulated by aging temperature and duration [12]. Notably, the relationship between IMF and shear force is not inherently deterministic; although they often covary negatively, this association can be attenuated or reversed depending on the relative contributions of structural, metabolic, and post-mortem factors, which differ across breeds, muscle types, and processing conditions [13]. Understanding the biological determinants of IMF and shear force is therefore of both scientific and economic importance.

The Songlei black pig is a synthetic line currently under development. It was established through a two-step crossbreeding scheme. First, Songliao black boars were crossed with Leixiang sows to produce F1 hybrids. Second, F1 sows were backcrossed with Songliao black boars. The resulting offspring were then intercrossed to stabilize desirable traits. The line is currently in its second generation of selection and has not yet been formally recognized as a breed. This breeding strategy combines the growth performance of the Songliao parent with the superior meat quality of the Leixiang parent. The DLY pigs used in this study were produced from a standard three-way cross (Duroc sires × Landrace × Yorkshire dams) and obtained from the same commercial breeding scheme to ensure genetic and management consistency.

Our previous phenotypic analyses revealed that Songlei black (SL) pigs exhibit significantly higher intramuscular fat content and lower shear force values compared with DLY pigs. Further integrated analysis of transcriptome and metabolome identified a set of differentially expressed genes and metabolites related to meat quality traits. These findings suggest that pathways such as lipid metabolism and muscle contraction may be involved. However, mRNA expression is tightly regulated by upstream non-coding RNAs. Therefore, analysis based solely on transcriptome and metabolome data is insufficient to reveal the complete regulatory mechanism [14,15,16]. To fill this gap, we turned our attention to non-coding RNAs. These molecules act as key regulators of gene expression at the post-transcriptional level.

Among them, microRNAs (miRNAs) are a class of endogenous, small non-coding RNA molecules. They are approximately 21–23 nucleotides in length and evolutionarily highly conserved [17]. miRNAs negatively regulate gene expression by binding complementarily to the 3′-untranslated region (3′-UTR) of target genes, which inhibits translation or promotes mRNA degradation [18]. It is estimated that more than 60% of protein-coding genes are regulated by miRNAs [19]. miRNAs participate in various biological processes, including cell proliferation, differentiation, and apoptosis. In recent years, they have also attracted considerable attention in the fields of muscle development and fat metabolism [20,21]. For example, muscle-specific miRNAs (myomiRs), such as miR-1 and miR-206, promote myoblast differentiation by targeting PAX7 [22]. In addition, miR-143 has been confirmed as a pro-adipogenic factor and is significantly upregulated during preadipocyte differentiation [23]. However, no studies have yet investigated miRNA expression profiles in the longissimus dorsi muscle of SL versus DLY pigs, nor have they explored miRNA-mediated regulatory networks potentially associated with their meat quality differences. In this study, we used small RNA sequencing to compare miRNA expression profiles in the longissimus dorsi muscle of SL and DLY pigs. We identified differentially expressed miRNAs between the two breeds by small RNA sequencing and further predicted their target mRNA interactions through bioinformatics analysis. A potential regulatory network was then constructed. In this study, we therefore set out to identify miRNA candidates potentially involved in the meat quality differences between SL and DLY pigs. The innovation of this study lies in the first comprehensive comparison of miRNA expression profiles in the longissimus dorsi muscle between a newly developed Chinese indigenous synthetic line (SL) and a commercial crossbreed (DLY), with systematic integration of miRNA-mRNA regulatory networks and correlation with IMF and shear force. We hypothesized that breed-specific differences in miRNA expression contribute to the distinct IMF and shear force phenotypes of SL and DLY pigs.

## 2. Materials and Methods

### 2.1. Animals and Sample Collection

Systematic phenotyping of meat quality traits in SL and DLY pigs was previously performed by our group (*n* = 40 per breed; data not published). Based on these results, six individuals per breed with intramuscular fat content closest to the breed mean were selected. This selection strategy was designed to minimize within-group phenotypic variability and ensure that the sequenced samples were representative of each breed‘s typical meat quality phenotype, thereby reducing the risk that observed differences in miRNA expression are driven by extreme individual variation rather than breed-specific biology. The sample size (*n* = 6 per breed) followed the recommendation of RNA-seq methodological studies, which indicate that six to seven biological replicates are sufficient to achieve reliable differential expression detection at log_2_FC ≥ 1 and *p* < 0.05. All experimental animals were obtained from Feimasi Animal Husbandry Co., Ltd. (Changchun, China) with both sexes included as they represent the normal commercial pig population. They were reared under identical housing and management conditions and fed the same commercial diets throughout the production cycle. SL and DLY pigs were housed in the same barn but in different pens under identical environmental conditions, with the same ventilation, temperature, lighting, and feeding systems. The individual animal was considered the experimental unit. Pigs were slaughtered at a target live weight of approximately 120 kg. All pigs were slaughtered in May. After 12 h of fasting with free access to water, pigs were stunned by electrical stunning, exsanguinated, dehaired, eviscerated, and split down the midline. Longissimus dorsi muscle samples were collected immediately after slaughter, snap-frozen in liquid nitrogen, and stored at −80 °C until analysis [24].

### 2.2. Determination of Intramuscular Fat and Shear Force

For IMF determination, visible fat, connective tissue, and intermuscular fat were removed, and the samples were minced. Approximately 100 g of minced meat was dried at 65 °C to constant weight, ground into powder, and dried again at 105 °C for 3 h. The dried powder was subjected to Soxhlet extraction with diethyl ether for 6–7 h. IMF content was calculated based on weight difference and expressed as a percentage of dry matter. Each sample was analyzed in triplicate. Shear force was measured according to the Chinese agricultural industry standard NY/T 1180-2006 [25]. Longissimus dorsi muscle samples were trimmed of visible fat and placed in plastic bags. The samples were aged at 4 °C for 24 h. The samples were then equilibrated at room temperature for 1 h, and a thermometer was inserted into the center of each sample. The bags were placed in a water bath at 80 °C until the internal temperature reached 70 °C. The samples were then removed and cooled to room temperature (approximately 20 °C). Cylindrical cores (1.27 cm in diameter, approximately 2.5 cm in length) were taken parallel to the muscle fiber direction using a round core sampler, with sampling locations at least 5 mm from the sample edge and at least 5 mm apart, according to NY/T 1180-2006. Shear force was measured using a tenderness meter (Bulader, Beijing, China) equipped with a Warner-Bratzler blade, with the muscle fiber direction perpendicular to the blade, at a test speed of 1 mm/s. Ten replicates were measured per sample and averaged. Results were expressed in Newtons (N).

### 2.3. Small RNA Library Construction and Sequencing

Total RNA was extracted from longissimus dorsi muscle samples using TRIzol reagent (Invitrogen, Carlsbad, CA, USA). RNA concentration and purity were measured using a Nanodrop 2000 (Thermo Scientific, Waltham, MA, USA). RNA integrity was assessed using an Agilent 2100 Bioanalyzer (Agilent, Santa Clara, CA, USA). Each animal gave rise to an independent sequencing library; no pooling was applied. Approximately 1 µg of total RNA was used per library. RIN values ranged from 8.2 to 9.2 (Table S1). RNA concentrations ranged from 92 to 204 ng/µL. Total RNA with an RNA integrity number (RIN) greater than 8.0 was used for small RNA library construction. Library preparation was randomized across groups, and all samples were processed in a single sequencing run to avoid batch effects. Libraries were prepared using TruSeqTM Small RNA Sample Prep Kits (Illumina, San Diego, CA, USA). miRNA library construction was based on the structural features of small RNA molecules, including a 3′ hydroxyl group and a 5′ phosphate group. The 3′ and 5′ adapters were ligated sequentially. First-strand cDNA was then synthesized using reverse transcription primers. Double-stranded cDNA libraries were obtained by PCR amplification. After purification, library fragments with insert sizes of 18–40 bp were selected. Qualified libraries were sequenced on an Illumina NovaSeq 6000 platform (Illumina, San Diego, CA, USA) (single-end, 50 bp). Adapter sequences were removed using fastp (v0.23.4). Average read yield was 11.2 million reads per sample (range: 9.8–13.4 million). Sequencing was completed by Novogene Technology Co., Ltd. (Beijing, China).

### 2.4. Sequencing Data Quality Control and Preprocessing

Raw sequencing data were saved in FASTQ format [26]. Data filtering was performed by the sequencing provider (Novogene Technology Co., Ltd.) using custom Perl and Python scripts. The filtering steps were as follows: (1) remove reads contaminated with 3′ adapters; (2) remove reads with more than 10% N bases; (3) remove reads with more than 50% low-quality bases (Q < 5). After filtering, high-quality clean reads were obtained. Subsequently, reads with lengths between 18 and 40 nt were selected for subsequent miRNA identification and analysis. The retention rate after filtering for each sample is presented in Table S5.

### 2.5. miRNA Identification

Clean reads, after length filtering, were mapped to the pig reference genome (Sscrofa11.1) using Bowtie version (v1.3.1) with no mismatches permitted, and multi-mapped reads were discarded. The genome and annotation files were downloaded from the Ensembl database [27]. Based on the alignment results, known miRNAs were identified by comparison with the miRBase database (version 22). The sequence, length, and expression abundance of known miRNAs in each sample were recorded [28]. Reads not annotated as known miRNAs were then aligned to non-coding RNA sequences (rRNA, tRNA, snRNA, snoRNA) in the Rfam database (version 13.0) to annotate other ncRNAs [29]. Unannotated reads were further aligned to a repeat sequence database to remove reads originating from repetitive elements. The remaining reads were mapped to exon and intron positions to annotate small RNAs derived from mRNA degradation fragments. Finally, novel miRNAs were predicted using miRDeep2 (v2.0.1.3) and srna-tools-cli software (v4.7) based on the hairpin structure characteristics of miRNA precursors. Novel miRNAs were classified based on the following criteria: minimum miRDeep2 score ≥ 1; presence of a stable hairpin secondary structure; minimum read count ≥ 10. The nomenclature for novel miRNAs was standardized as novel_XXX (e.g., novel_494). The precursor sequences of all novel miRNAs have been provided in Supplementary Table S6. The 191 novel miRNAs were subjected to additional filtering based on read count (≥10 reads in at least one sample) and structural criteria (presence of hairpin structure and miRNA) before inclusion in the final dataset. The sequence, length, and expression information of novel miRNAs in each sample were obtained [28,30].

### 2.6. Screening of Differentially Expressed miRNAs and Validation of Sequencing Results

miRNA expression levels were normalized to TPM (Transcripts Per Million reads) [31]. Differential expression analysis was performed using DESeq2 (v1.42.0) with raw read counts as input, and the Benjamini–Hochberg method was used for multiple testing correction. Differentially expressed miRNAs (DEMs) were identified using DESeq2 (v1.42.0) with |log_2_FC| ≥ 1 and padj < 0.05 as the thresholds [32]. Low-abundance miRNAs were not pre-filtered prior to DESeq2 analysis. Principal component analysis (PCA) was performed to evaluate the overall expression profiles and to detect potential outliers. To validate the sequencing data, eight DEMs were randomly selected for RT-qPCR. Specific primers were designed with Primer 5 based on the polyadenylation principle (Table S2). U6 served as the endogenous control, and relative expression was calculated via the 2^−ΔΔCt^ method. Each sample was tested in triplicate. Primers for qRT-PCR were synthesized by Genewiz.

### 2.7. Target Gene Prediction and Functional Enrichment Analysis of Differentially Expressed miRNAs

miRNA target genes were predicted using miRanda (miRanda-3.3a) and RNAhybrid (v2.0) software. The intersection of the predictions from both tools was taken as the final target gene set. Gene Ontology (GO) and Kyoto Encyclopedia of Genes and Genomes (KEGG) enrichment analyses were performed on the target genes of differentially expressed miRNAs using clusterProfiler (v4.8.1). An adjusted padj < 0.05 was considered the threshold for significant enrichment [33].

### 2.8. Construction of the Visualization of the miRNA-mRNA Regulatory Network

To construct the miRNA-mRNA regulatory network, small RNA sequencing data from this study were integrated with mRNA expression profiles obtained from transcriptome sequencing of the same 12 animals. Whole-transcriptome sequencing of the 12 samples yielded 168.08 Gb of raw data and 164.45 Gb of clean data after quality control. Reads were normalized using FPKM (Fragments Per Kilobase of transcript per Million mapped reads), and differentially expressed mRNAs were defined as |log_2_FC| ≥ 1 and padj < 0.05. The transcriptomic data have been deposited in the Genome Sequence Archive under accession number CRA037333 (Table S3). Pearson correlation coefficients were calculated for each miRNA-mRNA pair. For each animal, miRNA and mRNA expression profiles were paired based on sample identity. First, negatively correlated pairs with correlation coefficients less than −0.8 and padj < 0.05 were selected. Subsequently, from these negative pairs, those in which both the miRNA and the mRNA were differentially expressed (padj < 0.05) were further screened. A regulatory pair was included in the network only if it simultaneously met the following criteria: (1) the miRNA was predicted to target the mRNA by both miRanda and RNAhybrid; (2) both the miRNA and the mRNA were differentially expressed between SL and DLY (padj < 0.05); and (3) the miRNA-mRNA expression pair showed a negative Pearson correlation (r < −0.8, *p* < 0.05). PCA and Spearman correlation analyses were additionally performed to assess sample clustering and the robustness of the correlations. Network visualization was performed using Cytoscape software (v3.8.2) [34].

### 2.9. Statistical Analysis

Data analysis and graphing were performed using Microsoft Excel and GraphPad Prism 10 software packages [35,36]. Data are expressed as mean ± SD. One-way ANOVA, followed by Tukey’s test, was used for multiple comparisons. Significance was set at *p* < 0.05, and *p* < 0.01 was considered highly significant.

## 3. Results

### 3.1. Comparison of Intramuscular Fat and Shear Force

Intramuscular fat (IMF) and shear force are important indicators for evaluating meat quality. Significant differences (*p* < 0.01) were observed between Songlei black (SL) pigs and Duroc × Landrace × Yorkshire (DLY) pigs in both IMF content and shear force in the longissimus dorsi muscle. Specifically, SL pigs had higher IMF content but lower shear force values (Table 1 and Table S4).

### 3.2. miRNA Sequencing and Analysis

**Figure 1:**
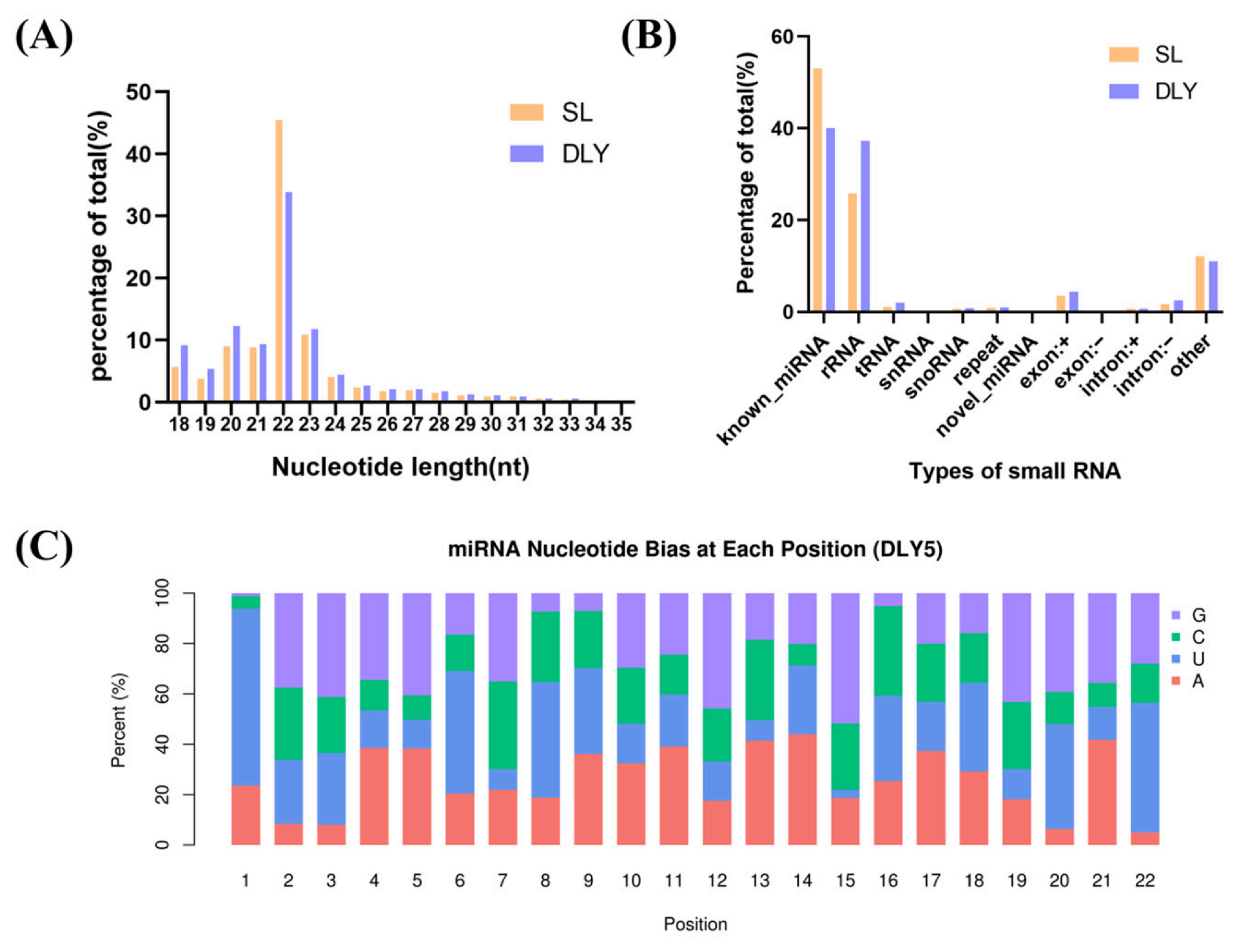
Expression of miRNAs in Songlei black (SL) and Duroc × Landrace × Yorkshire (DLY) pigs. (**A**) Length distribution of miRNAs in SL and DLY pigs. (**B**) Distribution of small RNA types. (**C**) Analysis of miRNA base preference.

Small RNA sequencing was performed on the longissimus dorsi muscle of SL and DLY pigs. A total of 70,271,997 and 64,636,243 raw reads were obtained from the two breeds, respectively. After removing adapter sequences and low-quality fragments, 68,401,486 and 62,590,870 high-quality clean reads were obtained, respectively (Table S5). The clean reads were aligned to the pig reference genome, with alignment rates of 96.24% and 95.50%, respectively. Among the obtained reads, most small RNAs ranged in length from 18 to 24 nt. Among these, 22-nt reads were the most abundant, accounting for 45.49% and 33.88% of total reads in SL pigs and DLY pigs, respectively (Figure 1A).

All small RNAs were annotated and classified according to the following priority: known miRNA > rRNA > tRNA > snRNA > snoRNA > repeat > gene > novel miRNA. Results showed that known miRNAs were the most abundant category. They accounted for 53.07% and 40.02% of total small RNA reads in SL pigs and DLY pigs, respectively (Figure 1B). In addition, the first nucleotide of mature miRNA sequences showed a clear bias, with uracil (U) being the most frequent (Figure 1C). This feature is consistent with the typical characteristics of miRNAs, indicating that the sequencing data were of reliable quality.

**Figure 2:**
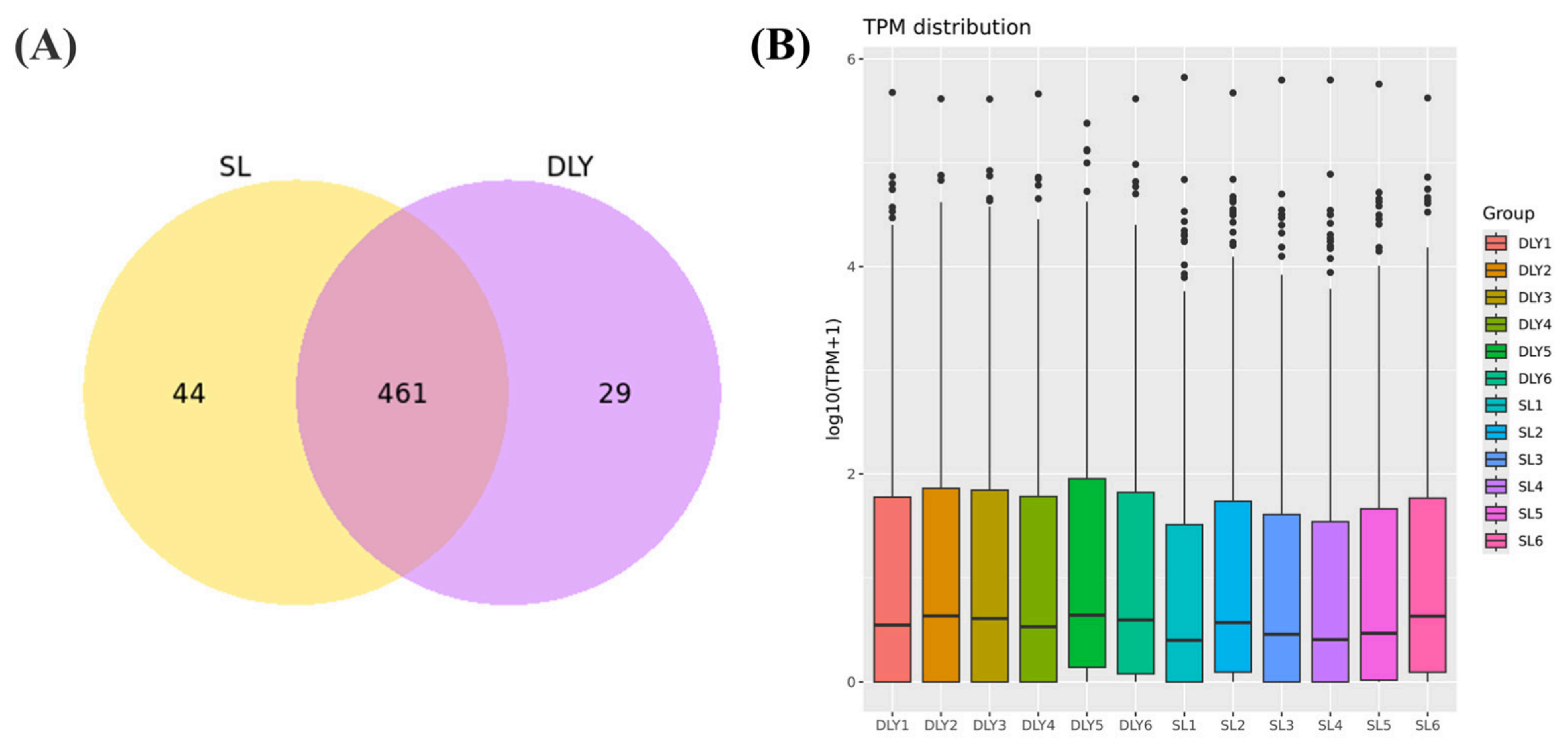
Expression levels of miRNAs in Songlei black (SL) and Duroc × Landrace × Yorkshire (DLY) pigs. (**A**) Venn diagram of co-expressed miRNAs. (**B**) Distribution of miRNA expression levels.

Through comparison with the miRBase database, 343 known mature miRNAs were identified in the two pig breeds, and 191 novel miRNAs were discovered (Table S6). Among these, 461 miRNAs were co-expressed in both breeds. Forty-four miRNAs were expressed only in SL, and 29 miRNAs were expressed only in DLY (Figure 2A). Within-sample gene expression distribution analysis showed that miRNA expression levels were relatively uniform between the two groups (Figure 2B). Among all identified miRNAs, ssc-miR-1 was the most abundant, with TPM values of 564,335 and 402,206 in SL and DLY, respectively. The next most abundant miRNAs were ssc-miR-206, ssc-miR-378, ssc-miR-133a-3p, and ssc-miR-486 (Table S7).

PCA based on miRNA expression profiles revealed clear separation between SL and DLY groups along PC1 (27.79% of variance) and PC2 (17.46% of variance), with no outlier samples detected (Figure S1). Spearman correlation analysis of miRNA expression profiles among all 12 samples revealed high consistency (Rho = 0.89–0.95) within and between groups, with no outlier samples detected (Table S8).

### 3.3. Screening and Validation of Differentially Expressed miRNA

**Figure 3:**
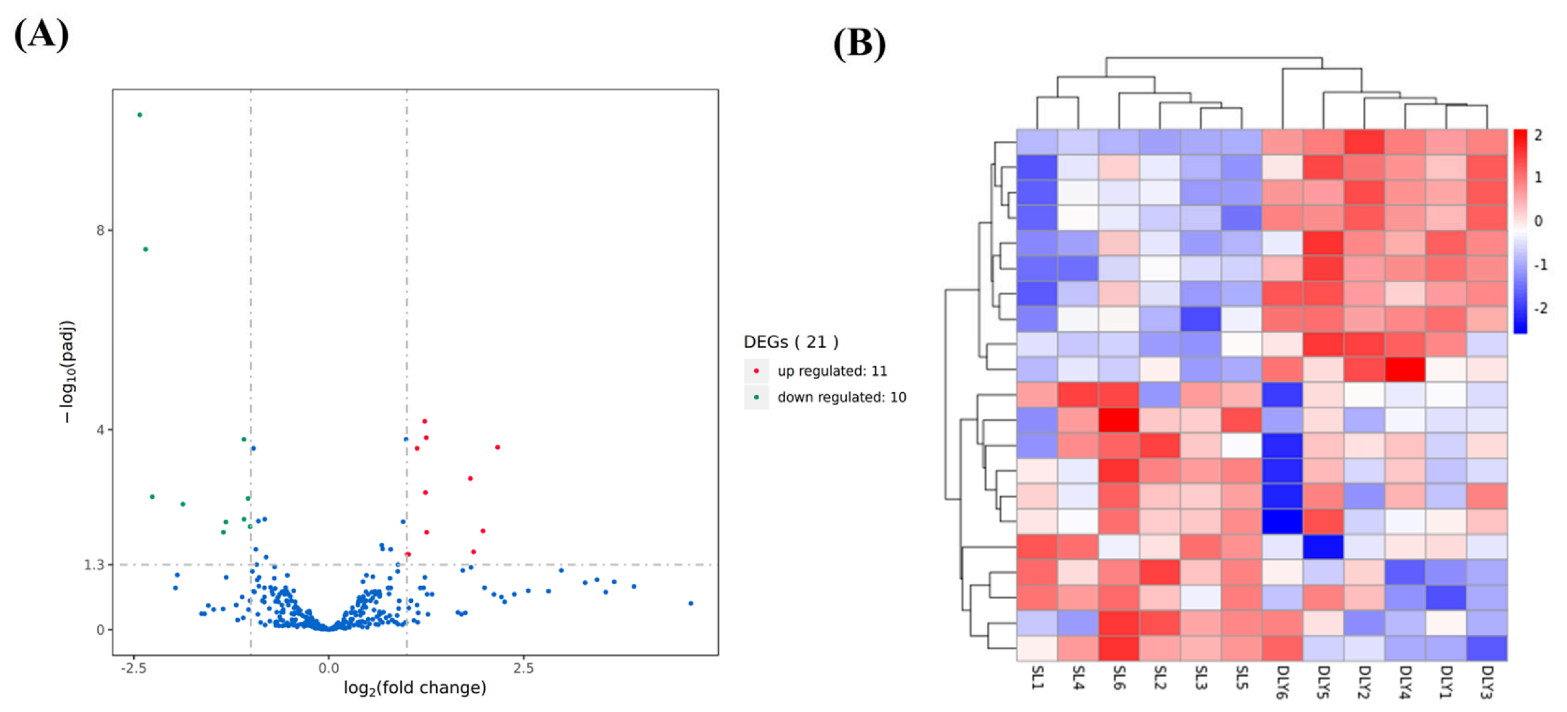
Differentially expressed miRNAs (DEMs) in the longissimus dorsi muscle of Songlei black (SL) and Duroc × Landrace × Yorkshire (DLY) pigs. (**A**) Volcano plot. Red and green dots indicate upregulated and downregulated miRNAs, respectively (*padj* < 0.05); blue dots: non-significant miRNAs. The dashed line marks the fold-change threshold. (**B**) Heatmap of DEMs.

**Figure 4:**
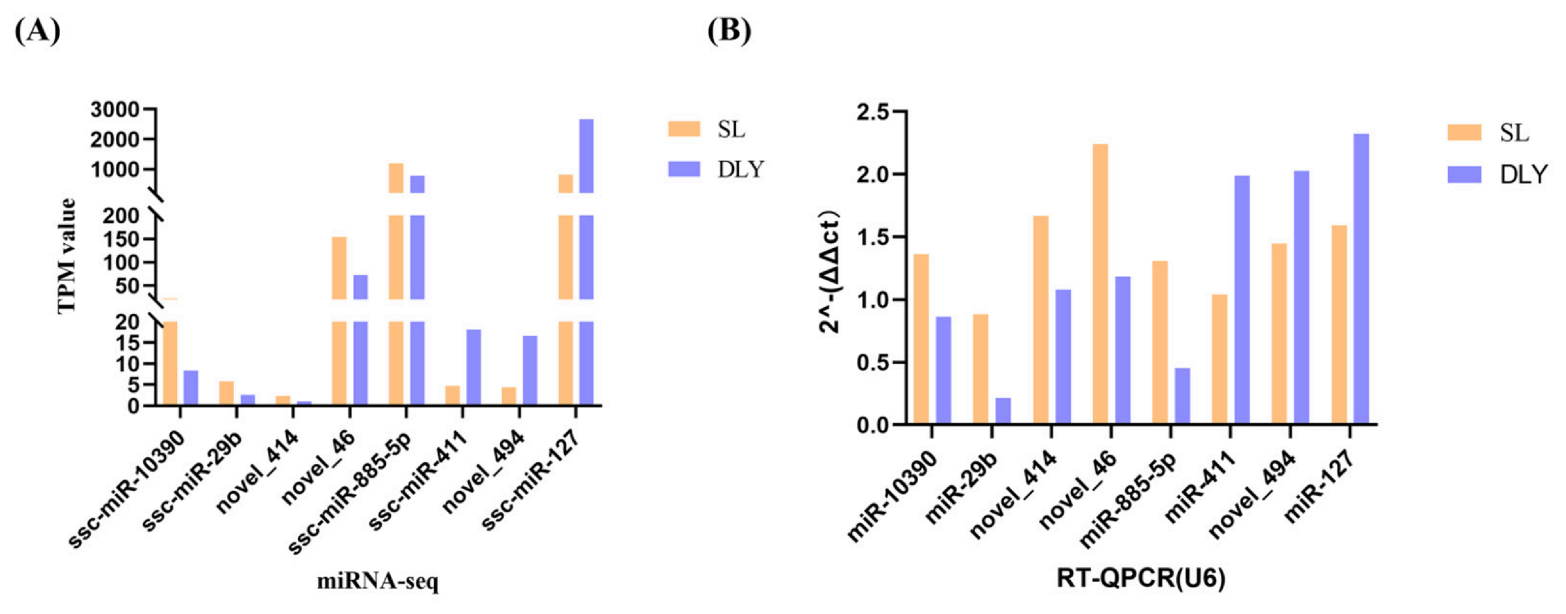
Comparison of miRNA expression levels using RNA sequencing (RNA-Seq) and reverse transcription quantitative polymerase chain reaction (RT-qPCR). (**A**) RNA-Seq expression profiles of selected miRNAs (TPM values). (**B**) RT-qPCR validation results (relative expression levels, 2^−ΔΔCt^ method). The expression trends between the two methods were consistent, confirming the reliability of the sequencing data. Abbreviations: TPM, transcripts per million; SL, Songlei black pigs; DLY, Duroc × Landrace × Yorkshire pigs.

Using |log_2_FC| ≥ 1 and padj < 0.05 as thresholds, a total of 21 differentially expressed miRNAs were identified between SL and DLY pigs. Of these, 11 miRNAs were upregulated in SL (7 known and 4 novel), while 10 were downregulated (7 known and 3 novel). Among the upregulated miRNAs, ssc-miR-885-5p showed the most significant differential expression, followed by ssc-miR-10390, ssc-miR-29b, and ssc-miR-664-5p. Notably, the muscle-specific miRNA ssc-miR-1 was also significantly differentially expressed between the two breeds after FDR adjustment (padj = 0.0029). Among the downregulated miRNAs, ssc-miR-10383 was the most significant, followed by ssc-miR-874 and ssc-miR-127 (Figure 3A, Table S7). The heatmap showed a clear clustering pattern of differentially expressed miRNAs between the two groups (Figure 3B). RT-qPCR was used to validate 8 randomly selected differentially expressed miRNAs. The results showed that the expression trends obtained by RT-qPCR were consistent with those from small RNA sequencing (Figure 4), indicating good reproducibility and reliability of the sequencing data.

### 3.4. Target Gene Prediction and Pathway Enrichment Analysis of Differentially Expressed miRNAs

To explore the potential functions of differentially expressed miRNAs, target gene prediction was performed using miRanda and RNAhybrid software. The prediction included the 21 significantly differentially expressed miRNAs, plus the one most highly upregulated and the one most highly downregulated miRNA in SL. The intersection of the predictions from both tools was taken as the final target gene set. Among these, ssc-miR-10390, ssc-miR-1, and novel_83 had no predicted target genes, while ssc-miR-411 had only one predicted target (Table S9).

**Figure 5:**
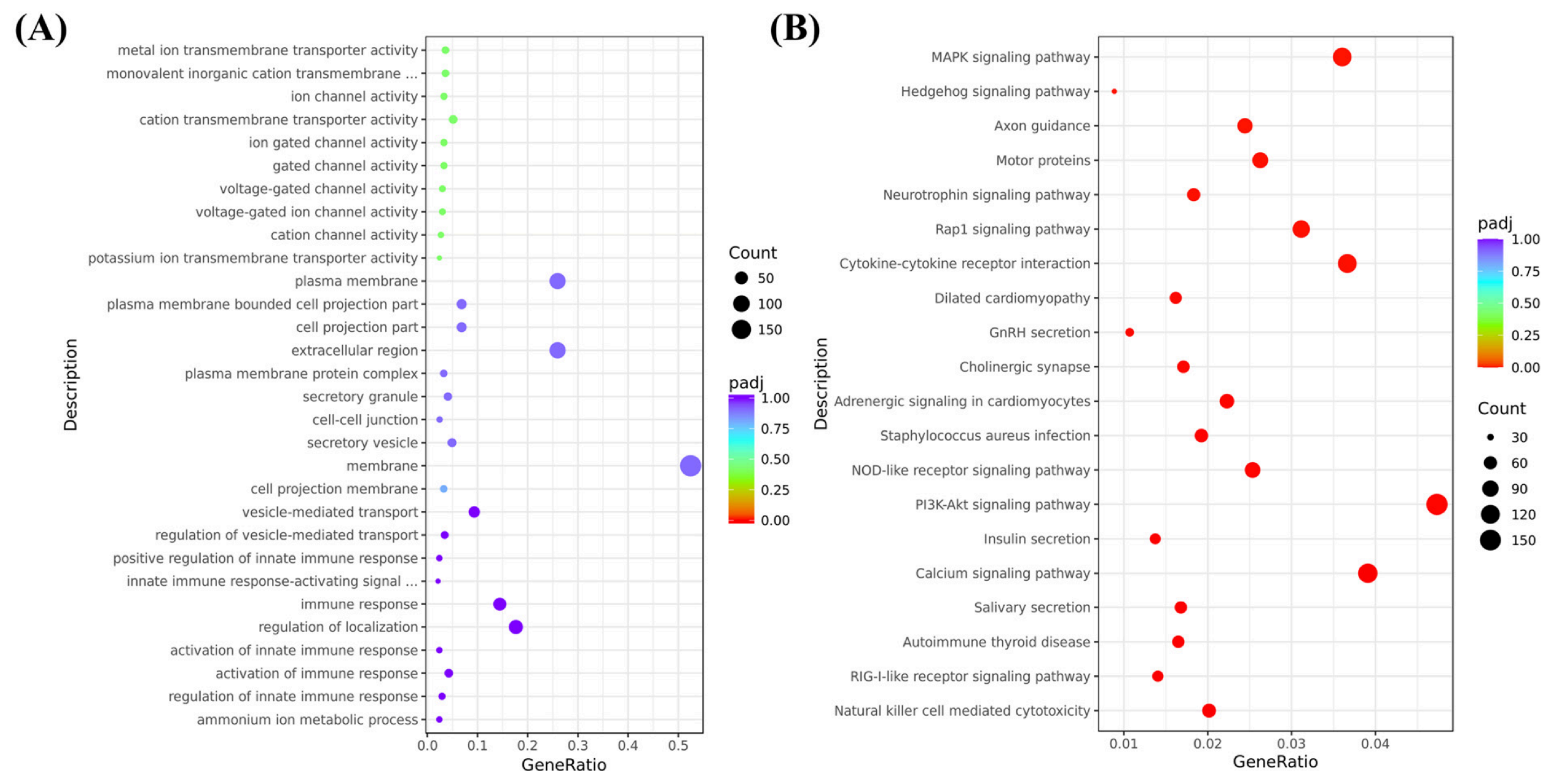
Enrichment analysis of miRNA target genes. (**A**) Gene Ontology (GO) enrichment. (**B**) Kyoto Encyclopedia of Genes and Genomes (KEGG) enrichment.

GO and KEGG enrichment analyses were performed on the target genes of 20 differentially expressed miRNAs (Figure 5, Table S10). GO enrichment analysis showed no significant enrichment at the threshold of padj < 0.05. However, enrichment trends were observed in plasma membrane structure, regulation of localization, and immune response. Specifically, the cellular component terms included plasma membrane, plasma membrane-bounded cell projection part, cell projection part, extracellular region, and membrane. The biological process terms included vesicle-mediated transport, immune response, and regulation of localization (Figure 5A). These results suggest that differentially expressed miRNAs may participate in the regulation of meat quality traits by modulating the above processes.

KEGG enrichment analysis identified 75 significantly enriched pathways. Among the top 20, PI3K-Akt, MAPK, and calcium signaling pathways were the main enriched terms, all of which are closely associated with muscle development and fat metabolism (Figure 5B).

### 3.5. Construction of the miRNA-mRNA Regulatory Network

**Figure 6:**
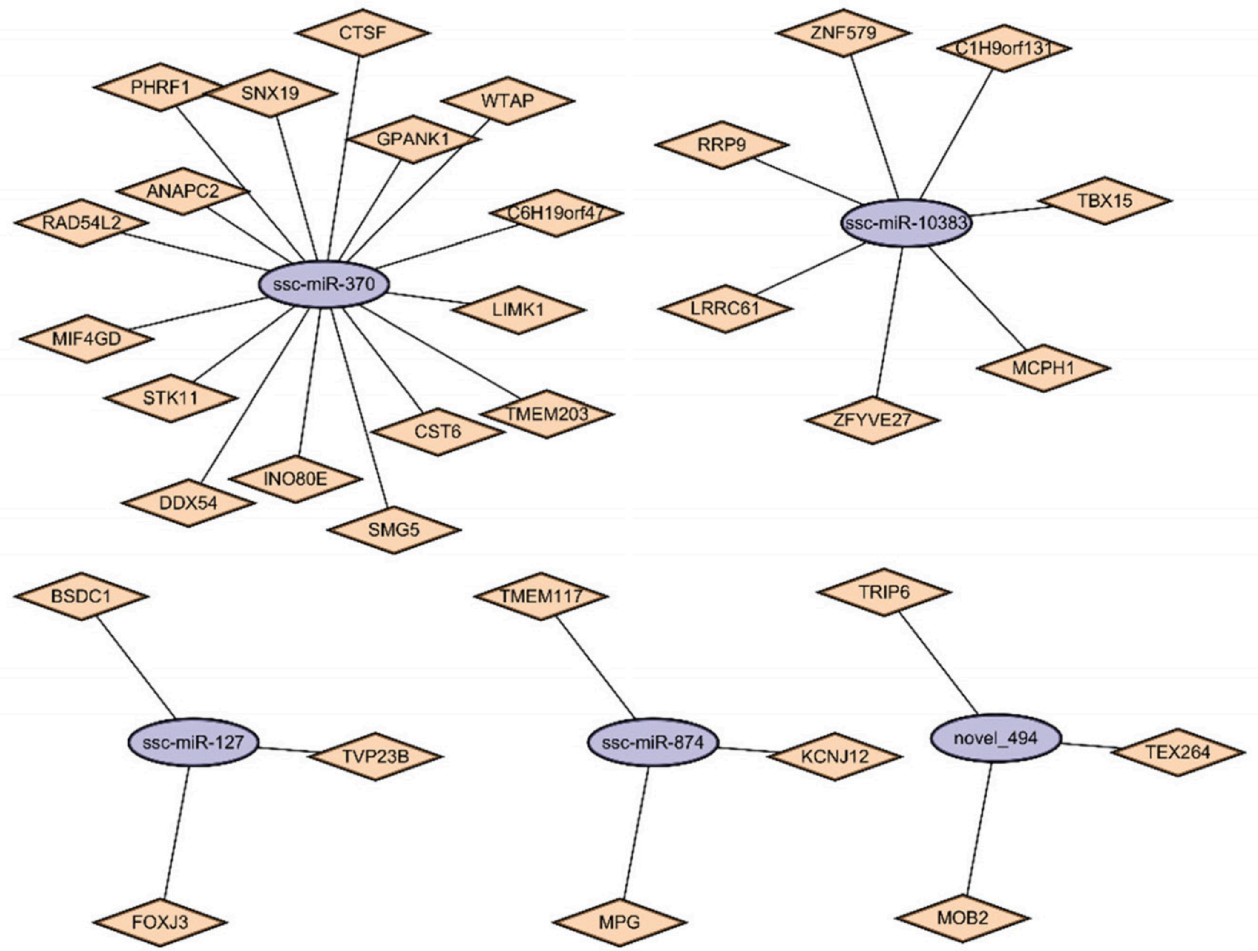
Interaction network of miRNA–mRNA.

To further identify specific miRNA-mRNA regulatory relationships, Pearson correlation analysis based on expression profiles was performed. A total of 32 miRNA-mRNA regulatory pairs (5 miRNAs and 32 mRNAs) were detected (Table S11, Figure 6). These 5 miRNAs included four known miRNAs (ssc-miR-10383, ssc-miR-127, ssc-miR-370, and ssc-miR-874) and one novel miRNA (novel_494).

### 3.6. KEGG Pathway Annotation of mRNAs in the miRNA-mRNA Network

**Figure 7:**
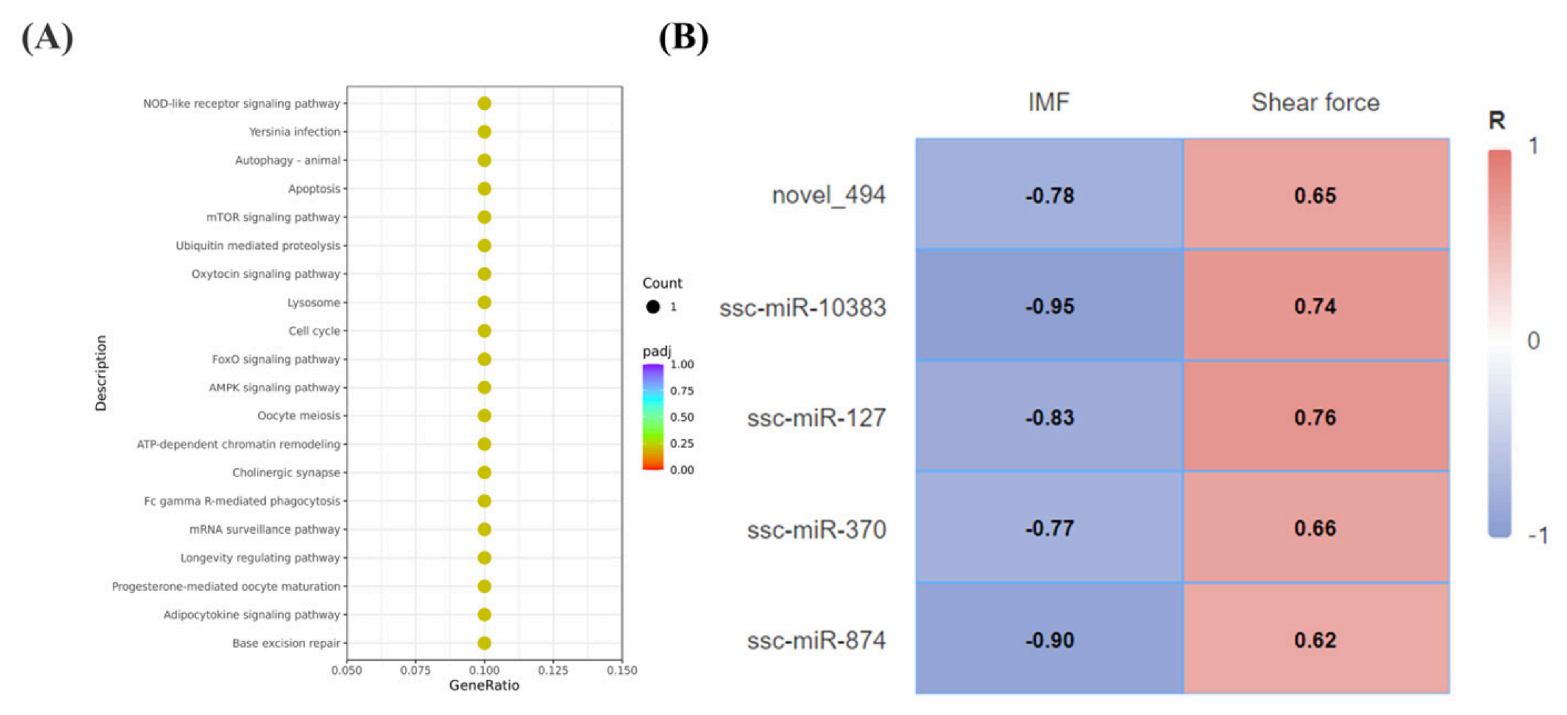
Functional enrichment and correlation analysis. (**A**) Kyoto Encyclopedia of Genes and Genomes (KEGG) pathway annotation of mRNAs in the miRNA-mRNA regulatory network. (**B**) Correlation analysis between differentially expressed miRNAs and intramuscular fat (IMF) content and tenderness. Pearson correlation coefficients and 95% confidence intervals are presented in Table S10.

To further characterize the biological functions of the mRNAs in the miRNA-mRNA regulatory network, we performed KEGG pathway annotation on the 32 mRNAs identified in the network (Figure 7A). These mRNAs were distributed across multiple pathways, including the NOD-like receptor, mTOR, AMPK, and FoxO signaling pathways, as well as autophagy and apoptosis. These pathways are closely related to cellular homeostasis, energy metabolism, and muscle physiology.

### 3.7. Correlation Analysis Between Differentially Expressed miRNAs and Intramuscular Fat and Shear Force

To further evaluate the association between the five identified miRNAs and meat quality traits, we analyzed their correlations with intramuscular fat (IMF) and shear force. The results showed that all five miRNAs were negatively correlated with IMF (r ranged from −0.77 to −0.95) and positively correlated with shear force (r ranged from 0.62 to 0.76), with 95% confidence intervals presented (Table S12). Among them, ssc-miR-10383 showed the strongest negative correlation with IMF (r = −0.95, 95% CI: −0.99 to −0.82), while ssc-miR-127 showed the strongest positive correlation with shear force (r = 0.76, 95% CI: 0.29 to 0.92) (Figure 7B). Additionally, ssc-miR-1 showed a correlation with IMF of r = 0.62 (95% CI: 0.39 to 0.88) and with shear force of r = −0.52 (95% CI: −0.84 to 0.06) (Table S12). These findings suggest that these miRNAs may be involved in regulating intramuscular fat deposition and muscle tenderness in pigs.

## 4. Discussion

The predominance of 22-nt reads and the uridine bias at the first nucleotide position are consistent with the typical features of Dicer-processed miRNAs [37] and with previous reports in skeletal muscle of other livestock species [38,39,40]. These observations collectively confirm the reliability of our sequencing data and the quality of the miRNA libraries [41].

In both breeds, the most highly expressed miRNAs were ssc-miR-1, ssc-miR-206, ssc-miR-378, ssc-miR 133a-3p, and ssc-miR-486. Among these, miR-1, miR-206, miR-133a-3p, and miR-486 are members of the myomiR family. MyomiRs have been reported as critical regulators of muscle fiber hypertrophy, regeneration, and satellite cell proliferation and differentiation [42]. miR-1 and miR-133a-3p are encoded by a bicistronic transcript and coordinately regulate myoblast differentiation and proliferation in skeletal muscle: miR-1 promotes differentiation, whereas miR-133 promotes proliferation [43]. miR-206 also promotes myoblast differentiation by targeting PAX7 and DNA polymerase alpha, as previously shown [44]. miR-486 is located within an intron of the ankyrin repeat domain containing protein 1 gene (ANK1) and promotes myoblast survival and differentiation by inhibiting PTEN and FoxO1 [45]. Although miR-378 is not a member of the myomiR family, it was highly expressed in skeletal muscle of both breeds. Previous studies have shown that it promotes myoblast differentiation and inhibits proliferation by targeting HDAC4 [46]. In the present study, all of the above miRNAs were highly expressed in both breeds. Notably, the expression level of ssc-miR-1 was higher in SL (TPM = 564,335) than in DLY (TPM = 402,206). This finding suggests that SL may have stronger myogenic transcriptional activity. MyomiRs not only regulate muscle growth but also indirectly influence fat infiltration [47,48,49]. Therefore, the higher ssc-miR1 expression in SL may be associated with the higher intramuscular fat content and lower shear force observed in this breed.

Although miRNAs are highly conserved across species, differential expressions were observed. A total of 21 differentially expressed miRNAs were identified between SL and DLY pigs. Among these, the most significantly upregulated miRNAs in SL were ssc-miR-885-5p, ssc-miR-10390, ssc-miR-29b, and ssc-miR-664-5p. The most significantly downregulated miRNAs in SL were ssc-miR-10383, ssc-miR-874, and ssc-miR-127. Among the upregulated miRNAs, ssc-miR-885-5p has been reported to promote myoblast proliferation and inhibit differentiation by targeting MyoD1 in bovine muscle [50]. This miRNA was upregulated in SL, which has high intramuscular fat and low shear force. This finding may reflect an active muscle growth state in SL, providing sufficient myofiber space for subsequent intramuscular fat deposition. In addition, it may be potentially associated with the low shear force of SL. ssc-miR-29b negatively regulates adipogenesis in pigs by targeting CTRP6 via the AKT/PKA/MAPK signaling pathway [51]. In the present study, miR-29b was upregulated in SL, which has high intramuscular fat. This result appears contradictory to the negative regulatory role of miR-29b in adipogenesis. Several non-mutually exclusive explanations may account for this discrepancy. First, miR-29b expression was measured in whole muscle tissue, which contains both myofibers and adipocytes, rather than in isolated adipocytes; its cell-type-specific effects may therefore differ. Second, the observed upregulation in muscle could represent a compensatory response aimed at counteracting excessive adipogenesis. Third, the upregulation of miR-29b in SL muscle tissue may also occur through indirect mechanisms involved in muscle–adipose crosstalk. Additionally, breed-specific (SL vs. DLY) differences in the regulatory network of miR-29b cannot be excluded. These possibilities warrant further investigation in purified cell populations. miR-664-5p shows significant differential expression in the longissimus dorsi muscle of Rongchang pigs. Direct targeting of the 3‘UTR of SRF and Wnt1 by this miRNA enhances cell cycle progression and represses myogenic markers (MyoG and MyHC) [52]. Hence, this miRNA is central to skeletal muscle myogenesis, as it promotes proliferation and represses differentiation. The high expression of ssc-miR-664-5p in SL reflects the strong proliferative activity of myoblasts in this breed, which lays a foundation for subsequent muscle development and intramuscular fat deposition. The biological function of ssc-miR-10390 remains insufficiently studied. The significance of its sustained upregulation in SL requires further exploration. Among the downregulated differentially expressed miRNAs, ssc-miR-10383 showed the most significant difference. Previous reports have indicated that high expression of ssc-miR-10383 inhibits UCP3 function and increases energy expenditure, which is unfavorable for intramuscular fat accumulation [53]. In the present study, ssc-miR-10383 was downregulated. This downregulation may relieve the inhibition of lipid synthesis related pathways, thereby promoting intramuscular fat deposition. Meanwhile, the significantly downregulated miRNAs ssc-miR-874 and ssc-miR-127 in SL deserve special attention. miR-874 has been confirmed as a candidate regulator of fat deposition in multiple studies. It acts as a core node in the regulatory network of intramuscular adipogenesis by targeting Wnt pathway genes such as PPARD, WNT10B, RSPO3, and WNT2B [54]. In addition, the expression quantitative trait loci (miR eQTL) of miR-874 are significantly co localized with traits including carcass fat percentage, backfat thickness, muscle pH, and cooking loss [55]. This finding confirms its multifaceted regulatory function in fat metabolism and meat quality formation. The significant downregulation of ssc-miR-874 in SL suggests that its low expression may relieve the inhibition of Wnt pathway target genes, thereby promoting the high intramuscular fat phenotype. miR-127 is highly enriched in skeletal muscle and its expression is upregulated during myoblast differentiation [56]. In this study, ssc-miR-127 showed significant downregulation in the low shear force muscle of SL. This finding suggests that its low expression may relieve the inhibition of myoblast differentiation, thereby promoting muscle development and myofiber maturation. This provides a molecular explanation for the meat tenderness advantage of SL. These DEMs may play important roles in the differences in intramuscular fat content and shear force between SL and DLY pigs.

It is known that miRNAs function by inhibiting their target genes. The target genes of DEMs were subjected to KEGG enrichment analysis. The results showed that these target genes were significantly enriched in the PI3K Akt, MAPK, and calcium signaling pathways. The PI3K Akt pathway plays a central role in insulin signaling and regulates protein synthesis, cell survival, and lipid metabolism. In muscle, activation of PI3K Akt promotes myofiber hypertrophy and inhibits protein degradation. In adipose tissue, this pathway promotes preadipocyte differentiation and lipid accumulation [57]. The MAPK pathway plays stage specific regulatory roles in skeletal muscle development. Activation of the ERK pathway generally enhances myoblast proliferation but blocks differentiation. By contrast, the p38 pathway plays a positive role in differentiation [58]. The calcium signaling pathway is critical for excitation contraction coupling. Calcium fluctuations regulate muscle fiber type and fatty acid oxidation through calcineurin and NFAT [59]. Overall, the DEMs identified in this study may coordinately regulate intramuscular fat deposition and tenderness in Songlei black pigs through the above pathways.

A miRNA-mRNA regulatory network was constructed based on expression correlation. This network contained 32 negative correlation pairs (r < −0.8, padj < 0.05), involving five miRNAs (ssc-miR-10383, ssc-miR-127, ssc-miR-370, ssc-miR-874, and novel_494) and 32 mRNAs. Pathway annotation of the mRNAs in this network revealed their involvement in multiple biologically relevant pathways, including the NOD-like receptor signaling pathway, autophagy, apoptosis, mTOR, AMPK, and FoxO signaling. The conversion of muscle to meat is closely linked to autophagy and apoptosis. Activation of the apoptotic pathway in post-mortem muscle is an important early event in meat tenderness development [60]. mTOR promotes lipid synthesis, whereas AMPK promotes fatty acid oxidation and inhibits lipid synthesis. Together, they coordinately regulate lipid metabolic balance [61]. FoxO transcription factors are involved in metabolic regulation in muscle and adipose tissue, including promoting lipid uptake and oxidation [62]. Collectively, the distinct regulation of these pathways in SL versus DLY pigs may account for the observed breed differences in meat quality at the molecular level.

Correlation analysis showed that all five miRNAs were negatively correlated with intramuscular fat (IMF) and positively correlated with shear force. This finding suggests that they collectively participate in regulating intramuscular fat deposition and muscle tenderness. miR-127 has been reported to inhibit adipogenesis in pigs. Overexpression of miR-127 reduces preadipocyte differentiation and triglyceride accumulation. In skeletal muscle, it also inhibits satellite cell proliferation and myogenesis [63]. In the present study, miR-127 was downregulated in SL, which has higher IMF, and was negatively correlated with IMF. This result is consistent with its function as an inhibitor of adipogenesis. miR-874 has been confirmed as a negative regulator of fat deposition in multiple mammalian species. It is downregulated in both bovine intramuscular fat and adipose-type porcine subcutaneous fat [64]. In this study, miR-874 was also significantly downregulated and strongly negatively correlated with IMF, which is consistent with the above reports. The function of ssc-miR-10383 has been only limitedly reported. However, existing studies suggest that it may affect energy metabolism and fat deposition by regulating UCP3 [53]. In this study, this miRNA showed the most significant downregulation and the strongest negative correlation with IMF. This finding implies that it is an important negative regulator of IMF deposition. novel_494 is a newly discovered miRNA in this study, and no functional reports are available for it. Nevertheless, its strong correlations with IMF and shear force make it a highly promising candidate regulatory factor. All five miRNAs were downregulated in SL, and most of them are inhibitors of adipogenesis. Their low expression may coordinately relieve the inhibition of adipogenesis-related target genes, thereby promoting higher IMF deposition in SL. In addition, some of these miRNAs are also involved in regulating muscle fiber type or muscle cell proliferation, which may indirectly affect tenderness. Overall, these molecules form a synergistic network that modulates meat quality traits.

Although the sample size (n = 6 per breed) is limited, it is comparable to that of similar miRNA-seq studies in livestock, and the selected animals were phenotypically representative of each breed. Nevertheless, several limitations should be acknowledged. First, the sample size may reduce the detection sensitivity for low-abundance miRNAs. Second, the functional roles of the identified miRNAs, particularly novel_494, warrant further validation in cellular and animal models. Third, the regulatory network was derived from correlation data and thus does not imply direct causality. Several directions merit further investigation. First, the regulatory roles of these five key miRNAs should be functionally validated via overexpression and knockdown experiments in myoblast and adipocyte models. Second, the sample size should be increased to verify the reliability of these candidate miRNA biomarkers. Third, the utility of these miRNAs as breeding markers for pork quality improvement warrants investigation. In addition, the cross-talk between these miRNAs and other non-coding RNAs, such as long non-coding RNAs and circular RNAs, deserves further study.

## 5. Conclusions

Using small RNA sequencing, we profiled miRNA expression in the longissimus dorsi muscle of SL and DLY pigs, identifying 21 DEMs and their enriched pathways. A regulatory network of five miRNAs and 32 mRNAs was constructed. These miRNAs were correlated with IMF and shear force, suggesting their involvement in meat quality regulation. Overall, this work provides candidate targets for molecular breeding of high-quality pork.

## Figures and Tables

**Table 1:** Comparison of intramuscular fat (IMF) content and shear force in longissimus dorsi muscle between SL and DLY pigs.

Items	SL ^1^	DLY ^1^	*p*
IMF, %	3.49 ± 0.10	2.28 ± 0.07	<0.001
Shear force, N	36.31 ± 8.23	56.76 ± 9.49	0.006

^1^ Songlei black pigs; DLY: Duroc × Landrace × Yorkshire pigs.

## Data Availability

The RNA-Seq raw data have been deposited in the Genome Sequence Archive of the National Genomics Data Center, China National Center for Bioinformation/Beijing Institute of Genomics, Chinese Academy of Sciences, under accession number CRA044296.

## Supplementary Materials

The following supporting information can be downloaded at https://www.mdpi.com/article/10.3390/foods15152693/s1: Table S1: RNA Sample Detection Report; Table S2: PCR primers used for RT-qPCR; Table S3: Differential expression mRNAs between SL and DLY; Table S4: IMF and shear force in longissimus dorsi muscle of SL and DLY; Table S5: Summary of data filtering; Table S6: The miRNAs detected in SL and DLY; Table S7: Differential expression analysis of miRNAs between SL and DLY; Table S8: Spearman correlation between samples; Table S9: Predicted target mRNAs of differentially expressed miRNAs; Table S10: Gene Ontology and KEGG pathway annotation of miRNA target genes in SL and DLY; Table S11: List of miRNA-mRNA target relationships. Table S12: Pearson correlation coefficients (r) and 95% confidence intervals between five key miRNAs and meat quality traits; Figure S1: PCA of miRNA expression profiles in SL and DLY pigs.

## Abbreviations

The following abbreviations are used in this manuscript:

miRNA	microRNA
DLY	Duroc × Landrace × Yorkshire
SL	Songlei black
IMF	Intramuscular fat
IACUC	Institutional Animal Care and Use Committee
TPM	Transcripts per million reads
KEGG	Kyoto Encyclopedia of Genes and Genomes
GO	Gene Ontology
