# Supplementary material for: Identification of Candidate miRNAs Associated with Shear Force and Intramuscular Fat in Pig Breeds
Source: Foods. 2026 Jul 30;15(15):2693. doi: 10.3390/foods15152693 (PMC13465267; doi:10.3390/foods15152693)
Supplement: Supplementary file 1 [file foods-15-02693-s001.zip › Figure S1 PCA of miRNA expression profiles in SL and DLY pigs.pdf]

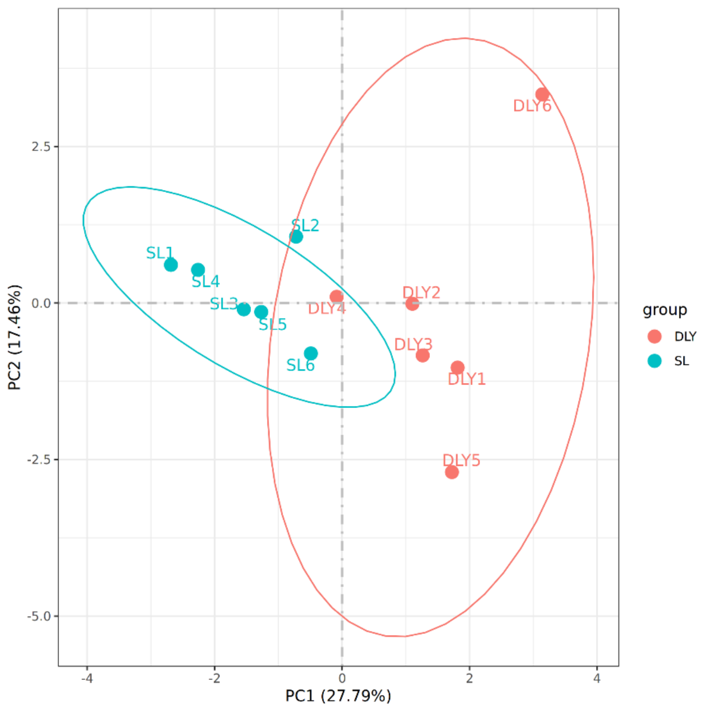

Figure S1 PCA of miRNA expression profiles in SL and DLY pigs
